# Ependymomas overexpress chemoresistance and DNA repair-related proteins

**DOI:** 10.18632/oncotarget.23288

**Published:** 2017-12-15

**Authors:** Sherise D. Ferguson, Shouhao Zhou, Joanne Xiu, Yuuri Hashimoto, Nader Sanai, Lyndon Kim, Santosh Kesari, John de Groot, David Spetzler, Amy B. Heimberger

**Affiliations:** ^1^ Department of Neurosurgery, The University of Texas MD Anderson Cancer Center, Houston, TX, USA; ^2^ Department of Biostatistics, The University of Texas MD Anderson Cancer Center, Houston, TX, USA; ^3^ Caris Life Sciences, Phoenix, AZ, USA; ^4^ Division of Neurosurgical Oncology, Barrow Neurological Institute, Phoenix, AZ, USA; ^5^ Department of Neurological Surgery and Medical Oncology, Thomas Jefferson University Hospital, Philadelphia, PA, USA; ^6^ Department of Translational Neurosciences and Neurotherapeutics, Pacific Neuroscience Institute and John Wayne Cancer Institute at Providence Saint John's Health Center, Santa Monica, CA, USA; ^7^ Department of Neuro-Oncology, The University of Texas MD Anderson Cancer Center, Houston, TX, USA

**Keywords:** ependymoma, chemoresistance, molecular profiling, DNA repair

## Abstract

**Background:**

After surgery and radiation, treatment options for ependymoma are few making recurrence a challenging issue. Specifically, the efficacy of chemotherapy at recurrence is limited. We performed molecular profiling on a cohort of ependymoma cases in order to uncover therapeutic targets and to elucidate the molecular mechanisms contributing to treatment resistance.

**Results:**

This ependymoma cohort showed minimal alterations in gene amplifications and mutations but had high expression rates of DNA synthesis and repair enzymes such as RRM1 (47%), ERCC1 (48%), TOPO1 (62%) and class III β-tublin (TUBB3) (57%), which are also all associated with chemoresistance. This cohort also had high expression rates of transporter proteins that mediate multi-drug resistance including BCRP (71%) and MRP1 (43%). Subgroup analyses showed that cranial ependymomas expressed the DNA synthesis enzyme TS significantly more frequently than spinal lesions did (57% versus 15%; *p* = 0.0328) and that increased TS expression was correlated with increased tumor grade (*p* = 0.0009). High-grade lesions were also significantly associated with elevated expression of TOP2A (*p* = 0.0092) and TUBB3 (*p* = 0.0157).

**Materials and Methods:**

We reviewed the characteristics of 41 ependymomas (21 cranial, 20 spinal; 8 grade I, 11 grade II, 22 grade III) that underwent multiplatform profiling with immunohistochemistry, next-generation sequencing, and *in situ* hybridization.

**Conclusions:**

Ependymomas are enriched with proteins involved in chemoresistance and in DNA synthesis and repair, which is consistent with the meager clinical effectiveness of conventional systemic therapy in ependymoma. Adjuvant therapies that combine conventional chemotherapy with the inhibition of chemoresistance-related proteins may represent a novel treatment paradigm for this difficult disease.

## INTRODUCTION

Ependymomas are glial tumors that can arise anywhere in the neuroaxis and account for approximately 4% of all malignant tumors in the central nervous system (CNS) [1]. They are postulated to originate from radial glia cells in the subventricular zone [2] and result in the development of tumors in three major anatomic compartments: the supratentorial cranium, the infratentorial cranium, and the spine. Ependymomas, which are more common in pediatric patients than in adult patients, constitute 6–12% of all childhood CNS neoplasms (1). The anatomical location of ependymomas largely depends on patient age, with 90% of pediatric patients having cranial lesions [3] and 60% of adult patients having tumors in the spine [4, 5]. Although ependymal tumors from different locations are histologically indistinct, their clinical behaviors are highly variable [6, 7]. Recent studies have identified ependymoma molecular subtypes that contribute to observed differences in clinical outcome [3, 8, 9].

Regardless of their subtype or location, the cornerstone of treatment for all ependymomas is surgery. Gross total resection (GTR) is well established to be associated with improved patient survival [7, 10], and spinal ependymomas can often be cured with complete resection alone [3, 11]. The GTR of cranial tumors located near eloquent structures (e.g., the fourth ventricle, the brainstem) can be challenging, and the potential for devastating neurological outcomes may preclude GTR in such cases [11, 12]. In both adult and pediatric ependymoma patients, maximal safe resection followed by adjuvant focal radiation is the standard of care. However, the role of adjuvant chemotherapy in the treatment of ependymal tumors is less established, and its overall benefit is controversial [13, 14].

Owing to insufficient adjuvant treatment options, patients with recurrent ependymoma face a devastating clinical scenario and poor survival [15–17]. Ependymomas are notoriously chemoresistant, which greatly limits options for salvage therapy. Only a few studies have investigated the mechanisms governing this chemoresistance [18–20]. Although recent breakthroughs have improved our understanding of the genetic and molecular characteristics of ependymomas [3, 8, 9, 21], the mediators of chemoresistance in ependymomas remain largely unknown [20, 22]. The purpose of the present study was to determine the distribution of chemoresistance-related proteins in ependymoma. We performed multi-platform profiling on a cohort of these tumors and found ependymomas are enriched with proteins critical for DNA synthesis and repair. These findings suggest new potential treatment approaches and may help guide the stratification of ependymoma patients in clinical trials of chemotherapy.

## RESULTS

### Patient and tumor characteristics

Patient and tumor characteristics observed were consistent with those in previous reports [11, 23]. Of the 41 ependymomas included in our analysis (20 [49%]) from males and 21 [51%] from females), 33 (80%) were adult and 8 (20%) were pediatric. Most ependymomas (26 [63%]) were cranial; 15 (37%) were spinal. Among the adult ependymomas, 19 (58%) were cranial and 14 (42%) were spinal; whereas among pediatric ependymomas, 7 (88%) were cranial and only 1 (13%) was spinal. The patient population was equally dichotomized based on gender with 49% being male (*n* = 20). Cranial tumors were more frequent in female patients (16/21 [76%]) than in male patients (10/20 [50%]). Tumor location was significantly correlated with patient age. Overall, the median age at which cranial tumors occurred (28 years) was significantly lower than that at which spinal tumors occurred (47 years; *p* = 0.008).

**Figure 1:**
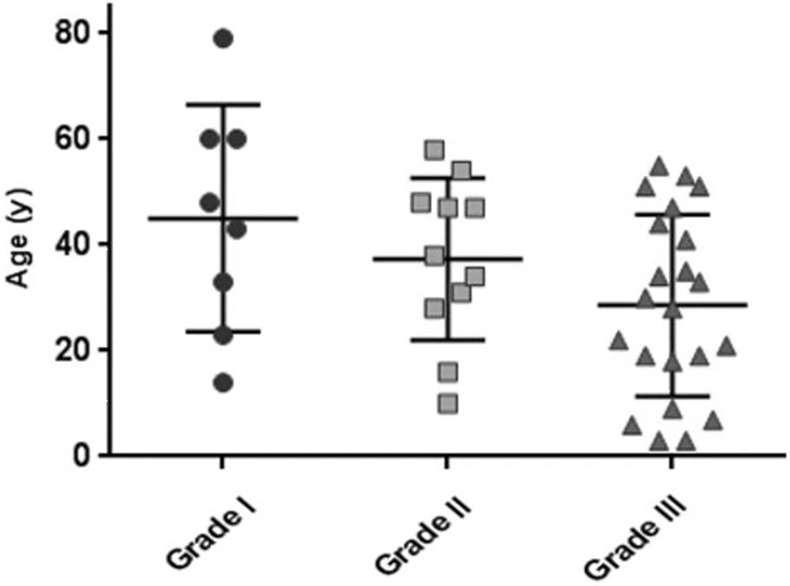
Scatterplot displaying the relationship between patient age and tumor grade

Eight patients (19%) had grade I tumors, 11 (27%) had grade II tumors, and 22 (54%) had grade III tumors. Tumor grade and patient age were significantly associated (Figure 1), which is consistent with a previous study's findings [24]. The median ages of patients with grade I, II, and III tumors (46, 38, and 29 years, respectively) differed significantly (*p* = 0.0213). There was also a relationship between tumor grade and location. Most grade I tumors were spinal (7/8 [88%]), whereas most grade II and III tumors were cranial (18/22 [82%]). All grade I spinal lesions were myxopapillary tumors. The percentages of cranial tumors that were grade I, II, or III (4% [1/26], 27% [7/26], and 69% [18/26], respectively) and those of spinal tumors that were grade I, II, or III (46% [7/15], 27% [4/15], and 27% [4/15], respectively) differed significantly (*p* = 0.0009).

### Chemoresistance proteins are highly expressed in ependymomas

**Figure 2:**
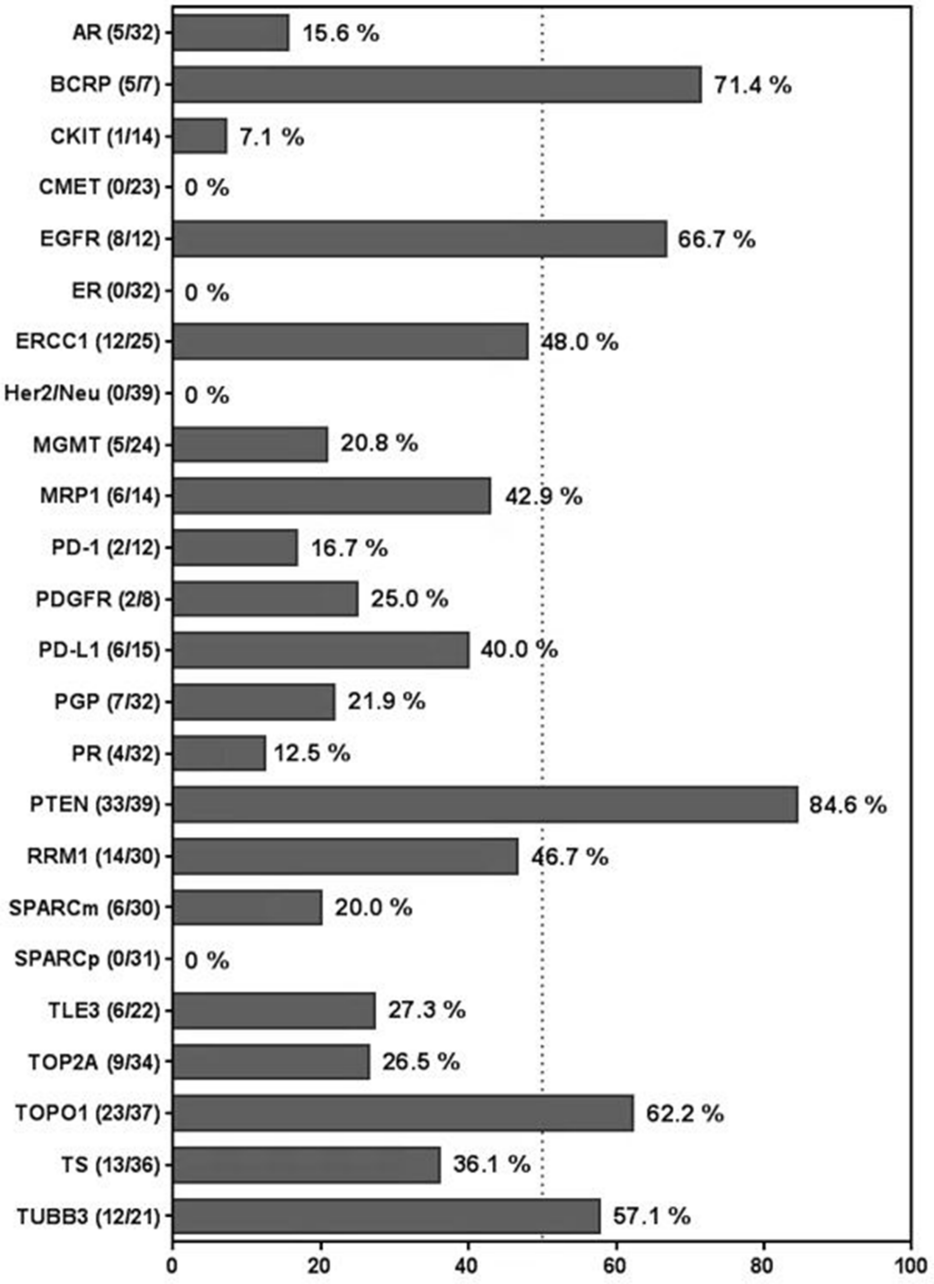
Bar graph displaying rates of cancer-associated protein overexpression among ependymomas

The rates of cancer-associated protein overexpression among ependymomas are given in Figure 2. Of the investigated proteins, PTEN was the most frequently expressed; specifically, 33 of 39 specimens (85%) showed PTEN overexpression. Five of 24 ependymomas (21%) had MGMT overexpression. Notably, ependymomas frequently overexpressed proteins implicated in DNA synthesis, transcription, and repair and/or drug resistance, including BCRP (5/7 [71%]), RRM1 (14/30 [47%]), ERCC1 (13/25 [48%]), TUBB3 (12/21 [57%]), TOPO1 (23/37 [62%]), and MRP1 (8/14 [43%]) [25–30]. Overall, 83% of the ependymomas overexpressed at least one DNA synthesis or repair protein or multi-drug resistance protein, and 80% overexpressed 2 or more.

### Chemoresistance protein expression depends on ependymoma grade and location

**Figure 3:**
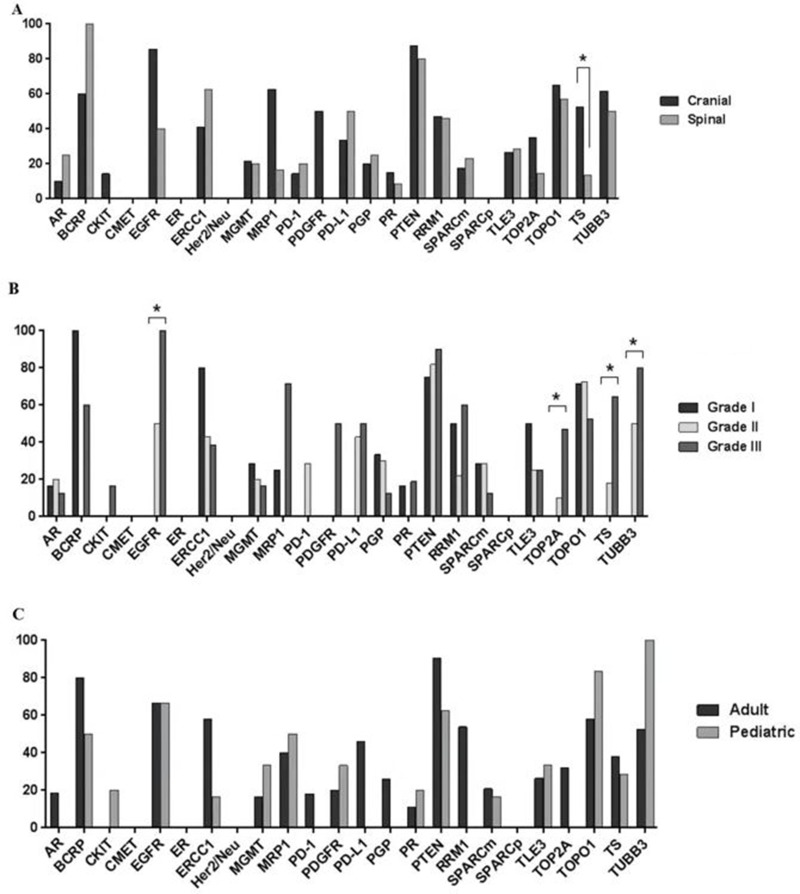
Bar graphs showing differential protein expression in ependymomas by (**A**) tumor location, (**B**) tumor grade, and (**C**) patient age. Asterisks indicate significant differences (*p* < 0.05)

The protein expression profiles of this ependymoma cohort by location, grade, and patient age are displayed in Figure 3. An analysis of the protein expression of 21 cranial and 15 spinal tumors revealed a significant association between TS expression and tumor location. The proportion of cranial ependymomas that overexpressed TS (57%) was significantly higher than that of spinal ependymomas that overexpressed the protein (15%; *p* = 0.0328) (Figure 3A). The proportion of cranial tumors that expressed EGFR and MRP1 was higher than that of spinal lesions; however, this difference was not statistically significant. In addition to its association with tumor location, TS overexpression was also correlated with tumor grade (Figure 3B). The frequency of TS overexpression among grade III tumors (65% [11/17]) was significantly higher than that among grade II tumors (18% [2/11]) and grade I tumors (0% [0/9]; *p* = 0.0009). Tumor grade was correlated with the overexpression of several additional proteins, including EGFR, which was overexpressed in 100%, 50%, and 0% of grade I, II, and III tumors, respectively (*p* = 0.0283); TOP2A, which was overexpressed in 47%, 10%, and 0% of grade I, II, and III tumors, respectively (*p* = 0.0092); and TUBB3, which was overexpressed in 80%, 50%, and 0% of grade I, II, and III tumors, respectively (*p* = 0.0157). Differences in protein expression between adult and pediatric cases did not differ significantly (Figure 3C).

### Cranial and spinal ependymomas have differential expression of chemoresistance proteins

**Figure 4:**
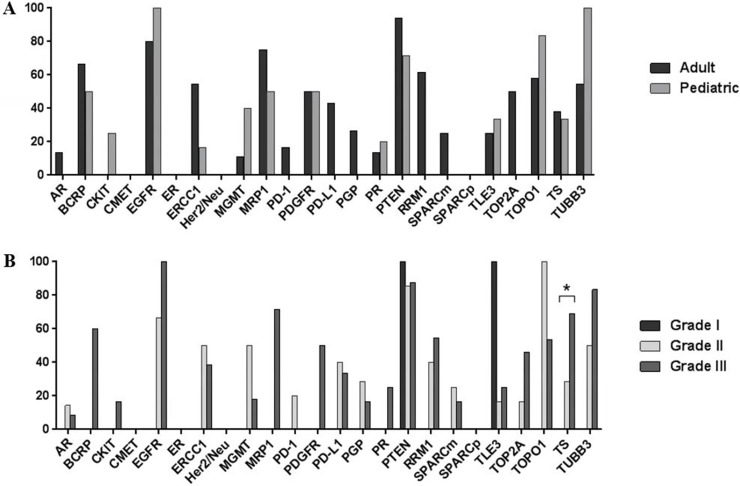
Bar graphs showing differential protein expression in cranial ependymomas by (**A**) patient age and (**B**) tumor grade. Asterisk indicates a significant difference (*p* < 0.05).

**Figure 5:**
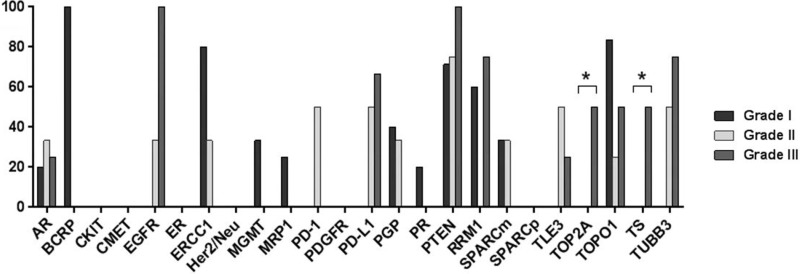
Bar graph showing differential protein expression in spinal ependymomas by tumor grade Asterisks indicate significant differences (*p* < 0.05).

A subgroup analysis of cranial ependymomas revealed that the biomarker profiles of adult and pediatric tumors did not differ significantly (Figure 4A). However, tumor grade and TS expression were significantly correlated, with 69% of grade III tumors displaying TS overexpression compared with 0% of grade I lesions (*p* = 0.042) (Figure 4B). In a subgroup analysis of spinal ependymomas, high tumor grade was associated with increased TS and TOP2A expression (Figure 5). TS was exclusively overexpressed in grade III tumors, with no TS expression detected in grade I or II tumors (*p* = 0.0286). TOP2A expression was observed in 50% of grade III spinal tumors and 0% of grade II or I tumors (*p* = 0.0361).

## DISCUSSION

We found that proteins involved in DNA synthesis (e.g. RRM1), transcription (TOPO1), and repair (ERCC1) are enriched in ependymoma. As these proteins affect DNA metabolism, several are key mediators of chemoresistance in other solid malignancies [26, 27, 29]. Specifically, the expressions of RRM1, ERCC1, and TUBB3 are inversely correlated with tumor responses to anti-metabolites such as gemcitabine, platinum-based agents, and spindle poisons, respectively, which may help explain why these drugs have shown little efficacy *in vivo* [31] or in clinical studies [32, 33]. We also found transporter proteins such as BCRP and MRP1, which have roles in multidrug resistance, to be elevated in our ependymoma cohort. BCRP (also referred to as ABCG2) belongs to the ATP-binding cassette transporter family and reduces the intracellular concentrations of multiple chemotherapeutic agents, including doxorubicin, topotecan, and mitoxantrone [34]. Our findings are consistent with those of a smaller study that reported that the BCRP and MRP1 genes are expressed in ependymomas [35]. BCRP-mediated multi-drug resistance may be reversed by EGFR tyrosine kinase inhibitors, and several preclinical studies (utilizing breast, colon and small cell lung cancer cell lines) have demonstrated this phenomenon [36–38]. Thus, the combination of transporter inhibition plus chemotherapy may be a novel treatment paradigm for refractory ependymoma [39].

Our subgroup analyses of cranial tumors and of spinal tumors demonstrated that ependymomas at different locations have differential protein expression. For example, although ependymomas had an overall low expression rate of TS, a key enzyme in DNA biosynthesis and a known marker of cell proliferation and poor prognosis in other malignancies [40], cranial ependymomas had a significantly higher expression rate of TS than spinal ependymomas did. This was not unexpected as cranial lesions are known to display more aggressive clinical behavior. Low TS expression is correlated with response to 5-fluorouracil [41], which indicates that that 5-fluorouracil–based therapy might be efficacious in a select cohort of ependymoma patients. In addition, commercially available direct TS inhibitors such as pemetrexed have shown efficacy in other solid malignances [42] and have yielded favorable results in patients with non–small cell lung cancer brain metastases in phase II trials [43, 44] and may hold promise for ependymoma patients [45].

We also found that the expression of TS, TUBB3, and TOP2A were associated with tumor grade. These proteins are associated with aggressive behavior, poor patient prognosis, and chemoresistance in other malignancies [27, 40, 46], and our results indicate a similar pattern in ependymoma. Elevated TOP2A expression in ependymoma has not been reported previously; however, etoposide, a TOP2A inhibitor, has been used to treat recurrent ependymoma with very modest clinical results, likely because the agent has meager CNS penetration [47–49]. WP744 (berubicin), another TOP2A inhibitor able to penetrate the blood-brain barrier, has been tested in primary CNS malignancies with some success; however, additional studies of this agent in ependymoma may be warranted [50].

Even though this study is strengthened by a wide array of biomarkers analyzed, it is limited by the fact that it is commercial database, hence clinical data regarding patient outcome is not available. In addition, data regarding the molecular subtype (i.e. RELA and/or YAP1 fusion) of these ependymoma samples was unavailable for the majority of cases as physicians rarely requested this specific testing. Moreover, the effect of previous treatments on protein expression cannot be analyzed.

## MATERIALS AND METHODS

### Study population

We reviewed the Caris Life Sciences database and identified 41 ependymal tumors submitted to the company between 2009 and 2015 for multiplatform analysis (including mutational analysis, *in situ* hybridization, and immunohistochemistry) for biomarker profiling. WHO criteria were used to grade tumors. Because Caris Life Sciences maintains a commercial database, specimens are not clinically annotated; thus, outcome (e.g., survival) and prior treatment data were not available for the present study. Biomarkers analyzed varied by case owing to the preference of the ordering physician, tissue availability, and variation in technology over the study period. This study was exempt from Institutional Review Board approval as per 45 CFR 46.101(b), as the data analyzed were from an existing commercial repository and patients’ protected health information was de-identified.

### Mutational analysis

Genomic DNA isolated from formalin-fixed, paraffin-embedded tumor tissue was sequenced with the Illumina MiSeq platform. The specific regions of 47 pan-cancer genes considered to be of interest and related to cancer genomics on the basis of current literature were amplified using the customized Illumina TruSeq Amplicon Cancer Hotspot panel. The hotspot regions of the following genes were sequenced: ABL1, AKT1, ALK, APC, ATM, BRAF, BRCA1, BRCA2, CDH1, cKIT, cMET, CSF1R, CTNNB1, EGFR, ERBB2, ERBB4, FBXW7, FGFR1, FGFR2, FLT3, GNA11, GNAS, GNAQ, HNF1A, HRAS, IDH1, JAK2, JAK3, KDR (VEGFR2), KRAS, MLH1, MPL, NOTCH1, NPM1, NRAS, PDGFRα, PIK3CA, PTEN, PTPN11, RB1, RET, SMAD4, SMARCB1, SMO, STK11, TP53, and VHL. All variants reported were detected with > 99% confidence based on the mutation frequency.

### *In situ* hybridization

Fluorescence *in situ* hybridization (FISH) and chromogenic *in situ* hybridization were used to detect amplification of cMET (with the cMET/CEP7 probe and a Ventana kit) and HER2 (with the HER-2/CEP17 probe and INFORM HER-2 Dual ISH DNA Probe Cocktail). FISH was also used to detect EGFR amplification (with the EGFR/CEP7 probe). cMET was considered to be amplified if ≥ 5 copies per tumor cell were detected on average. A HER-2/CEP17 ratio of ≥ 2 was considered to indicate amplified HER2. EGFR amplification was defined by the presence of an EGFR/CEP7 ratio of ≥ 2 or ≥ 15 EGFR copies per cell in ≥ 10% of analyzed cells.

### Immunohistochemistry

Formalin-fixed, paraffin-embedded tumor specimens were used for the immunohistochemical analysis. Antibodies against the following proteins were used: androgen receptor (AR), cMET, cKIT, epidermal growth factor receptor (EGFR), estrogen receptor (ER), excision repair cross-complementation group 1 (ERCC1), human epidermal growth factor receptor 2 (HER2), O(6)-methylguanine-methyltransferase (MGMT), p-glycoprotein (PGP), programmed cell death protein 1 (PD-1), programmed death-ligand 1 (PD-L1), platelet-derived growth factor receptor alpha (PDGFR), phosphatase and tensin homolog (PTEN), progesterone receptor (PR), ribonucleotide reductase M1 (RRM1), SPARC (monoclonal and polyclonal), thymidylate synthase (TS), topoisomerases 1 and 2 (TOPO1, TOP2A), transducin-like enhancer of split 3 (TLE3), and tubulin beta-3 chain (TUBB3). The primary antibody clones are listed in Supplementary Table 1. The conditions for staining were implemented using automated staining techniques and in accordance with the manufacturer's instructions. Staining conditions were validated following the requirements of the Clinical Laboratory Improvement Amendments/Compliance Assistance Office and International Organization for Standardization. Immunohistochemical staining scores were based on staining percentage (0–100%) and intensity (0 = no staining; 1+ = weak staining; 2+ = moderate staining; 3+ = strong staining). Independent board-certified pathologists confirmed the results. PD-L1 staining was specific to membranous tumor cells, and PD-1 staining was specific to tumor-infiltrating lymphocytes.

### Statistical analysis

Fisher's exact test, Cochran-Mantel-Haenszel chi-squared test and Cochran Armitage test were used to assess differences in biomarker expression rates between groups. All analyses were exploratory, performed with R v3.3.1 with package DescTools v0.99.16. *P* values ≤0.05 were defined as significant.

## CONCLUSIONS

Our findings demonstrate that chemoresistance-related proteins are markedly upregulated in ependymomas, and large-scale studies are needed to determine the extent to which this expression pattern is related to patient outcomes. However, our data suggest that a more tailored treatment approach based on biomarker expression may be warranted to better stratify ependymoma patients for specific therapies and clinical trials. Furthermore, an adjuvant treatment that combines conventional chemotherapy with drugs that inhibit DNA repair–related proteins or ATP-binding cassette transporter proteins may have enhanced clinical efficacy in appropriately selected patients.

## SUPPLEMENTARY MATERIALS TABLE


