# Supplementary material for: Ependymomas overexpress chemoresistance and DNA repair-related proteins
Source: Oncotarget. 2017 Dec 15;9(8):7822–31. doi: 10.18632/oncotarget.23288 (PMC5814261; doi:10.18632/oncotarget.23288)
Supplement: Supplementary file 1 [file oncotarget-09-7822-s001.pdf]

Ependymomas overexpress chemoresistance and DNA repair-related proteins

SUPPLEMENTARY MATERIALS

Supplementary Table 1: Primary antibody clones for IHC

Protein	Primary Antibody (Manufacturer, Catalog #)
AR	AR27 (Leica, NCL-AR-318)
PR	1E2/100 (Ventana, 790–4296)
ER	SP1 (Ventana, 790–4325)
HER2	4B5 (Ventana, 790–2991)
EGFR	31G7 (Zymed, Invitrogen, 28–0005
cMET	SP44 (Ventana, 790–4430)
TOPO1	1D6 (Leica, NCL-TOPO1)
TOPO2A	3F6 (Leica, NCL-TOPOIIA)
TS	TS106/4H4B1 (Invitrogen, 18–0405)
TUBB3	Polyclonal (Covance, PRB-435P)
PGP	C494 (Invitrogen, 18–7243)
MGMT	MT23.3 (Invitrogen, 18–7337)
PTEN	6H2.1 (DAKO, M3627)
TL3	Polyclonal (Sigma, HPA054116)
RRM1	Polyclonal (Proteintech, 10526–1-AP)
ERCC1	8F1 (Abcam, AB2356)
PD-1	MRQ-22 (Ventana, 760–4895)
PD-L1	130021 (R&D Systems, MAB1561)
SPARCm	12251 (R&D Systems, MAB941)
SPARCp	Polyclonal (EXALPHA, X1867P)
